# Exanthematic dengue fever mimicking rubella^☆^^☆☆^

**DOI:** 10.1016/j.abd.2020.06.006

**Published:** 2020-11-20

**Authors:** Dario Palhares

**Affiliations:** Universidade de Brasília, Brasília, DF, Brazil

**Keywords:** Dengue, Immunoglobulin E, Immunoglobulin G, Serology

## Abstract

The authors present a case of dengue fever mimicking rubella. Male patient, in the second episode of dengue fever, presented afebrile, with diffuse morbilliform rash and craniocaudal progression, having subsequently affected his palms and soles. On the third day of clinical evolution, serologies did not indicate IgM, IgG, or NS1, but on the sixth day of evolution, IgM and IgG were reactive for dengue fever. Previous episodes of dengue are a risk factor for the development of more severe conditions, but this was atypical because the patient was afebrile and had a rubelliform rash. The case also illustrates an early IgG anamnestic response, as it was a reinfection.

## Introduction

Dengue fever is a systemic infectious disease transmitted by mosquitoes of the genus Aedes, caused by the dengue virus, which presents with five serotypes (serotypes 1 to 4 are observed worldwide and serotype 5 has been identified in India).^1^ Infection by one of the serotypes does not confer immunity against the others.^1^ Risk factors for the development of severe dengue conditions include: previous episode of dengue, advanced age, and presence of comorbidities.^2^, ^3^, ^4^

## Case report

**Figure 1:**
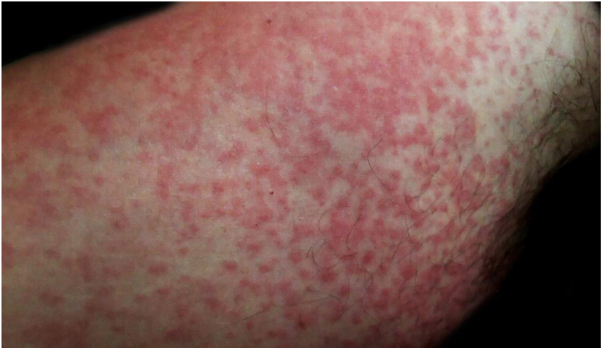
Detail of the rash. Overview of the rash.

**Figure 2:**
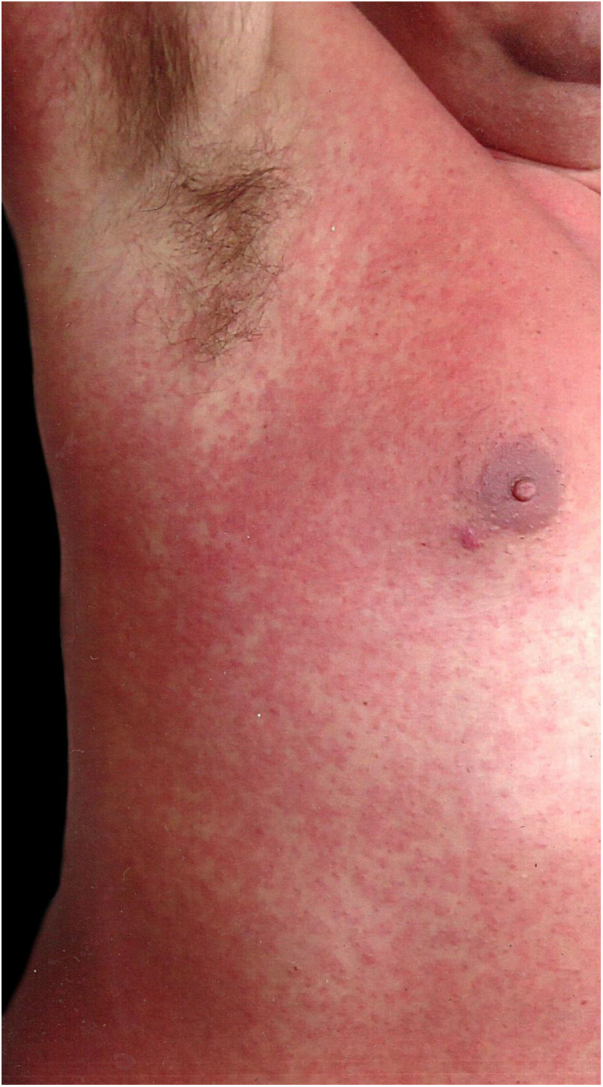
Overview of the rash. Rubelliform rash, on the third day of clinical evolution. The right axillary region was spared.

**Figure 3:**
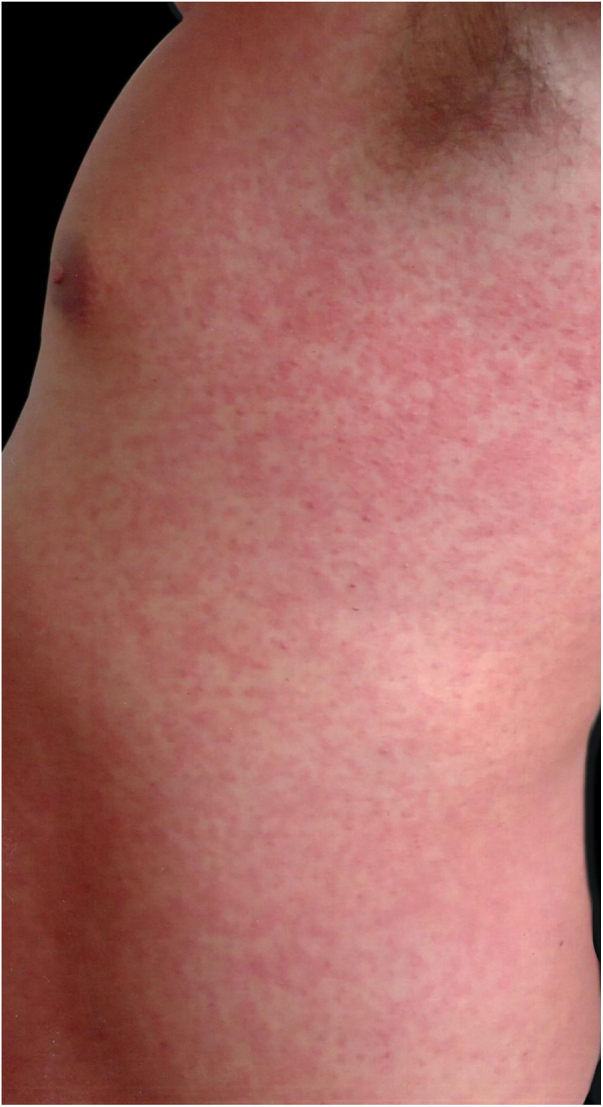
Third day of clinical evolution, left side of the body: the axillary region was affected.

A 42-year-old male patient, previously vaccinated for hepatitis A and B, had had first dengue infection 15 years earlier, with a febrile presentation and positive IgM serology. He reported that two days before presenting to this service, small papules appeared on the trunk, but that morning his body presented a rubelliform rash (Figs. 1–3) affecting from neck to knees and elbows, but sparing the face. He complained of asthenia but denied fever. The general physical examination showed no abnormalities other than the rash, not even palpable nodes. Complementary exams on arrival were as follows: blood count with hemoglobin, 15.4 g/dL; platelets, 170,000/mL; leukocytes, 4,020 mL (segmented 39%, eosinophils 3%, basophils 1%, lymphocytes 35%, monocytes 22%); IgM, IgG, and NS1 were negative for dengue fever; transaminases, bilirubin, and muscle enzymes were normal. The rash progressed two more days, affecting his palms and soles. The patient reported some diarrheal episodes, and denied fever. On the sixth day of evolution, the rash started to disappear, and the exams showed the following: hemogram with hemoglobin, 15.8 g/dL; platelets, 148,000/mL; and leukocytes, 4,910 mL (segmented 20%, eosinophils 5%, lymphocytes 59%, atypical lymphocytes 6%, monocytes 10%). Serologies: immune pattern for toxoplasmosis, cytomegalovirus, measles, herpes simplex, and Epstein-Barr virus. Non-reactive for HIV, syphilis, Zika virus, chikungunya, borreliosis, and rubella. IgG and IgM were positive for dengue fever. Total IgE was 713.8 IU/mL. The patient recovered completely and was revaccinated for rubella.

This case is unusual because it was a second episode of dengue, with afebrile presentation and mimicking rubella, with cranio-caudal progression of rubelliform rash. Over the years, IgG titers against dengue fever tend to fall and eventually become negative, and lower IgG titers are a protective factor against severe forms of dengue.^5^ However, in the case of a second infection, there is an anamnestic response, with early onset of IgG, and it is possible that an eventual third episode of dengue in this patient will be more severe. The NS1 antigen has a sensitivity of approximately 85% and tends to be lower in second episodes of dengue, *i.e*., in clinical practice a relatively considerable proportion of patients will not be diagnosed in the initial stage of the disease.^6^ It is possible that the patient's allergies have influenced the clinical presentation, with a rash that is not the classically described for dengue.^7^

Since the rash was spontaneously and quickly resolved, biopsies were not performed, as would be recommended if the rash became chronic. However, in this case, the blood count proved to be useful for clinical management, as it showed a typically viral pattern, even regarding atypical lymphocytes related to various diseases, including dengue fever and rubella.^8^, ^9^, ^10^ In conclusion, the relationship between parasites and hosts is complex, and dengue should be part of the differential diagnosis of rashes, even though the patient does not present classic symptoms, such as fever or orbital pain. Furthermore, in the emergency room setting, the complete blood count, an exam usually ready in a few hours, can assist in the clinical decision.

## Financial support

None declared.

## Authors’ contributions

Dario Palhares: Approval of the final version of the manuscript; design and planning of the study; drafting and editing of the manuscript; collection, analysis, and interpretation of data; critical review of the literature; critical review of the manuscript.

## Conflicts of interest

None declared.
